# Influenza vaccination coverage rates among adults before and after the 2009 influenza pandemic and the reasons for non-vaccination in Beijing, China: A cross-sectional study

**DOI:** 10.1186/1471-2458-13-636

**Published:** 2013-07-08

**Authors:** Shuangsheng Wu, Peng Yang, Haiyue Li, Chunna Ma, Yi Zhang, Quanyi Wang

**Affiliations:** 1 Beijing Center for Disease Prevention and Control, Institute for Infectious Disease and Endemic Disease Control, Beijing, China

**Keywords:** Influenza vaccine, Vaccination, Coverage, Adult

## Abstract

**Background:**

To optimize the vaccination coverage rates in the general population, the status of coverage rates and the reasons for non-vaccination need to be understood. Therefore, the objective of this study was to assess the changes in influenza vaccination coverage rates in the general population before and after the 2009 influenza pandemic (2008/2009, 2009/2010, and 2010/2011 seasons), and to determine the reasons for non-vaccination.

**Methods:**

In January 2011 we conducted a multi-stage sampling, retrospective, cross-sectional survey of individuals in Beijing who were ≥ 18 years of age using self-administered, anonymous questionnaires. The questionnaire consisted of three sections: demographics (gender, age, educational level, and residential district name); history of influenza vaccination in the 2008/2009, 2009/2010, and 2010/2011 seasons; and reasons for non-vaccination in all three seasons. The main outcome was the vaccination coverage rate and vaccination frequency. Differences among the subgroups were tested using a Pearson’s chi-square test. Multivariate logistic regression was used to determine possible determinants of influenza vaccination uptake.

**Results:**

A total of 13002 respondents completed the questionnaires. The vaccination coverage rates were 16.9% in 2008/2009, 21.8% in 2009/2010, and 16.7% in 2010/2011. Compared to 2008/2009 and 2010/2011, the higher rate in 2009/2010 was statistically significant (*χ*^2^=138.96, p<0.001), and no significant difference existed between 2008/2009 and 2010/2011 (*χ*^2^=1.296, p=0.255). Overall, 9.4% of the respondents received vaccinations in all three seasons, whereas 70% of the respondents did not get a vaccination during the same period. Based on multivariate analysis, older age and higher level of education were independently associated with increased odds of reporting vaccination in 2009/2010 and 2010/2011. Among participants who reported no influenza vaccinations over the previous three seasons, the most commonly reported reason for non-vaccination was ‘I don’t think I am very likely to catch the flu’ (49.3%).

**Conclusions:**

Within the general population of Beijing the vaccination coverage rates were relatively low and did not change significantly after the influenza pandemic. The perception of not expecting to contract influenza was the predominant barrier to influenza vaccination. Further measures are needed to improve influenza vaccination coverage.

## Background

Influenza is a major cause of global morbidity and mortality each year, especially in the elderly and those with chronic diseases [1-4]. Vaccination is an effective measure to reduce influenza-related morbidity and mortality [5-7]. To optimize the vaccination coverage rates in the general population, the status of coverage rates and the reasons for non-vaccination must be understood. Several studies focusing on seasonal influenza vaccination coverage rates have been conducted among specific groups, such as the elderly, patients with high-risk conditions, and healthcare workers [8-11]. These studies reported low uptake rates against seasonal influenza in high-risk populations. A fear of side effects, doubts about vaccine efficacy, concerns about the danger of influenza, perceived susceptibility to influenza, and information provided by healthcare professionals all influence the coverage rate of seasonal influenza vaccination [11]. Some general population-based surveys about vaccination coverage rates have been conducted [12,13]. Surveys from 5 European countries have shown consistently low coverage rates in the general population in 2006/07 (25.0% in UK, 27.4% in Germany, 21.8% in Spain, 24.2% in France, and 24.4% in Italy) [12,13]. Since 2007, seasonal influenza vaccination has been provided free of charge between September and November each year; the priority populations in Beijing include people ≥ 60 years of age and primary and middle school students. No general population-based surveys involving influenza vaccination coverage have been conducted since the policy of free seasonal vaccination was implemented in Beijing.

At the end of March 2009, an outbreak of influenza A (H1N1) infection occurred in Mexico, followed by a spread worldwide [14]. Vaccines based on the new virus have been rapidly developed, and H1N1 vaccination campaigns have been adopted in many countries; however, low acceptance of a vaccine or uptake rates against pandemic influenza were reported in many studies (25% among health workers in Beijing, 17.0% among a French adult population, and 8.9% among pregnant women in Turkey) [15-19]. A systematic review reported that many factors affect the uptake of pandemic vaccination, such as perceptions of personal risk and the severity of the pandemic, perceived efficacy of the vaccine, perceived barriers to having the vaccine, social influences, sources of information about vaccination, and demographic factors [20].

Beijing is the capital of the People’s Republic of China and is one of the most populous cities in the world, with a population of nearly 20 million as of 2010. Due to the large population and high residential density, residents in Beijing are highly susceptible to influenza. In Beijing, the first case of H1N1 infection was reported on 16 May 2009. As of 30 December 2009, 10802 cases of influenza were confirmed, including 621 severe cases and 73 deaths [21]. The Chinese government undertook a series of measures according to WHO guidelines, including free vaccinations against pandemic influenza to populations at high risk (e.g., the elderly, public servants in key positions, students, teachers, and people with chronic diseases) [22]. However, a previous study undertaken in seven urban and two rural areas of China estimated the uptake of seasonal influenza vaccine to be 7.5% and pandemic influenza to be 10.8% in 2009, and few residents (25.1%) worried about being infected by influenza A (H1N1) [22]. Understanding whether or not the pandemic affected the influenza vaccination coverage and the underlying reasons for non-vaccination may help decision-makers take appropriate measures to protect people against influenza infections.

The aim of this study was to estimate vaccination coverage rates in the general population of Beijing before and after the 2009 influenza pandemic using data from three influenza seasons (2008/2009, 2009/2010, and 2010/2011) and to identify possible demographic factors associated with uptake of the vaccine. We also sought to explore reasons for non-vaccination.

## Methods

### Participants

The target population was Chinese adults ≥ 18 years of age living in Beijing. The respondents were classified into four subgroups according to population density (urban or suburban) and gender (male or female). The respondents were classified as urban/suburban according to the district where the participants were recruited. The formula used to estimate the sample size in each subgroup was as follows: $N=\frac{\mu_{\alpha }^{2}\times \pi \left(1-\pi \right)}{\delta^{2}}\times \mathrm{deff}$, with an *a* error of 5%, an overall influenza vaccination coverage rate (*π*) of 20%, a permissible error (*δ*) of 0.1 *π*, and a hypothesis of design effect (*deff*) of 2. Therefore, a sample size of 12294 questionnaires was calculated to obtain accurate estimates for influenza vaccination coverage rates.

There are 16 districts in Beijing, which are divided into urban and suburban districts based on population density. The population density was > 6548 people per km^2^ in the urban districts and ≤ 1305.4 people per km^2^ in the suburban districts. We randomly selected three urban districts and three suburban districts from the 16 districts. The survey was undertaken in the six districts. Participants were recruited using a multi-stage cluster sampling technique in each district. In the first stage, five towns/streets per district were randomly selected. In the second stage, five communities in each of these towns/streets were randomly selected. In the third stage, households were randomly selected. All households were numbered according to the address numbers, and 29–43 households per community were randomly selected for interviews. The interviewers visited the households individually, and interviewed each adult within the households until 87 residents were investigated in each community. The number of adults surveyed per randomly-selected household ranged from 1 to 6, with a mean of 2.4 and a median of 2.

### Data collection

The retrospective cross-sectional survey was conducted in January 2011. The survey was carried out using a self-administered, anonymous questionnaire. If the respondents could not understand the questionnaires, the well-trained investigators with a bachelor’s degree in epidemiology would read and explain the questionnaires to the respondents. To obtain the highest possible response rates, most of the visits were undertaken by local health workers who had good relationships with the participants and knew how to motivate the participants. The interviewers would make an appointment before visiting a family. In addition, re-visits were made to homes where all residents were absent.

The questionnaire consisted of three sections: (1) demographics (gender, age, educational level, and residential district name); (2) history of influenza vaccination in the 2008/2009, 2009/2010, and 2010/2011 seasons; and (3) reasons for non-vaccination (listed in a fixed order as follows: ‘I have never considered it before;’ ‘I don’t think the vaccine is effective enough;’ ‘I don’t think I am very likely to catch the flu;’ ‘I don’t think influenza is a serious illness;’ ‘I am afraid of the side-effects;’ ‘I have the specific contraindications;’ ‘The influenza vaccination is too expensive;’ and ‘I have no time to get vaccination’). All the response options were based on evidence in the existing literature [11,20]. The respondents were allowed to state more than one reason for non-vaccination. The respondents vaccinated in all three seasons were not required to answer the question of reasons for non-vaccination.

### Ethics statement

This study was approved by the Institutional Review Board and Human Research Ethics Committee of Beijing Center for Disease Prevention and Control. At the beginning of each interview, the agreement and verbal consent of the interviewee was obtained. Anonymity of the participants was guaranteed.

### Statistical analysis

The main outcome was the vaccination coverage rate. The rate in 2009/2010 included both seasonal and pandemic influenza vaccinations, as both seasonal and pandemic influenza vaccination campaigns were conducted in this season. Weighted analysis was conducted to calculate the age, gender, and residence-specific vaccination rates and frequencies, accounting for the age, gender, and urban/suburban population distribution of the Beijing population, as reported in the 2010 Census of Beijing. The difference among the subgroups was tested using a Pearson’s chi-square test with a two-sided p value <0.05 considered to be statistically significant. Possible determinants of influenza vaccination uptake were investigated by multivariate logistic regression. Gender, age, educational level, and population density were included as independent variables. The multivariate model was conducted using a forward stepwise (Wald chi-square) method with a p value <0.05 for entry and a p value ≥ 0.10 for removal. Adjusted odds ratios (ORs) with 95% confidence intervals (CIs) evaluated the magnitude of the association between influenza vaccination and the demographics. All the statistical analyses were carried out using SPSS (version 13.0; SPSS, Inc., Chicago, IL, USA).

## Results

### Description of the sample

Among the 13287 questionnaires distributed in this survey, 13002 were completed and returned (response rate = 97.9%). Of the 13002 participants, 51.7% (n=6713) were female and 49.4% (n=6427) lived in an urban area. The near equal-sized samples in the urban and suburban areas were due to the sample design that three urban and three suburban districts were selected. The distribution of ages was as follows: 18–29 years, 20.7% (n=2697); 30–39 years, 19.5% (n=2540); 40–49 years, 20.0% (n=2602); 50–59 years, 20.3% (n=2642); and ≥ 60 years, 19.4% (n=2521). The distribution of educational levels was as follows: illiteracy, 3.0% (n=386); primary school, 10.8% (n=1409); junior high school, 28.2% (n=3664); senior high school, 28.4% (n=3692); and 3 year college graduate or above, 29.5% (n=3838). Age groups and gender were nearly equally distributed in each community/village.

### Influenza vaccination coverage rates

The vaccination coverage rates were 16.9% in 2008/2009, 21.8% in 2009/2010, and 16.7% in 2010/2011 (Table 1). The higher rate in 2009/2010 compared to 2008/2009 and 2010/2011 was statistically significant (*χ*^2^=138.96, p<0.001); no significant difference was found between 2008/2009 and 2010/2011 (*χ*^2^=1.296, p=0.255). Nonetheless, the vaccination coverage rates remained higher in 2010/2011 as compared to 2008/2009 among the elderly (36.3% vs. 43.1%, *χ*^2^=28.841, p<0.001). Overall, 9.4% of the respondents received vaccination in all three seasons, whereas 70% of the respondents did not receive any vaccinations during the same period (Table 2).

**Table 1:** Influenza vaccination coverage rates in 2008/2009, 2009/2010, and 2010/2011

	**season 2008/2009**	**season 2009/2010**	**season 2010/2011**
	**Weighted % (95% CI)**	**Weighted % (95% CI)**	**Weighted % (95% CI)**
Gender			
male	16.3 (15.3-17.2)	21.3 (20.2-22.3)	16.0 (15–16.9)
female	17.6 (16.7-18.5)	22.3 (21.3-23.3)	17.5 (16.5-18.4)
*χ*^2^	0.61	0.004	0.69
p value	0.437	0.953	0.407
Age (years)			
18~29	13.7 (12.4-15)	18.7 (17.2-20.1)	12.3 (11–13.5)
30~39	13 (11.7-14.3)	16 (14.6-17.4)	11.4 (10.2-12.6)
40~49	13.3 (12–14.6)	17.2 (15.6-18.6)	11.6 (10.4-12.8)
50~59	15.3(13.9-16.6)	19.5 (18–21)	14.9 (13.6-16.2)
≥60	36.3 (34.5-38.2)	46 (44.1-48)	43.1(41.1-45)
*χ*^2^	729.45	973.10	1340.82
p value	<0.001	<0.001	<0.001
Educational level			
illiteracy	32.9 (28.2-37.6)	43.3 (38.3-48.2)	40.4 (35.5-45.3)
primary school	24.6 (22.3-26.8)	33.7 (31.2-36.2)	30.7 (28.3-33.1)
junior high school	16.1 (14.9-17.3)	20.6 (19.3-21.9)	17.5 (16.3-18.8)
senior high school	15.5 (14.4-16.7)	20.0 (18.7-21.3)	15.2 (14.1-16.4)
3 year college graduate or above	18.5 (17.2-19.7)	23.5 (22.1-24.8)	16.2 (15–17.3)
*χ*^2^	123.62	208.65	303.16
p value	<0.001	<0.001	<0.001
Population density			
urban	17.7 (16.7-18.7)	22 (21–23)	16.6 (15.7-17.6)
suburban	15.6 (14.7-16.5)	21.5 (22.3-24.3)	16.8 (15.9-17.8)
*χ*^2^	7.96	0.01	0.537
p value	0.005	0.925	0.464
Total	16.9 (16.2-17.5)	21.8 (21.1-22.5)	16.7 (16.1-17.4)

**Table 2:** Frequency of influenza vaccination in 2008/2009, 2009/2010, and 2010/2011

	**Frequency of influenza vaccination Weighted % (95% CI)**				**χ**^**2**^	**p value**
	**0**	**1**	**2**	**3**		
Gender						
male	71.1 (70–72.2)	13.5 (12.7-14.3)	6.2 (5.6-6.8)	9.3 (8.5-10.1)	0.35	0.555
female	68.7 (67.6-69.8)	14.8 (14–15.6)	6.9 (6.3-7.5)	9.6 (8.9-10.3)		
Age (years)						
18~29	73 (71.3-74.7)	15.4 (14–16.8)	5.6 (4.7-6.5)	6.1 (5.2-7)	1453.47	<0.001
30~39	75.8 (74.1-77.5)	13.7 (12.4-15)	5 (4.2-5.8)	5.6 (4.7-6.5)		
40~49	74.9 (73.2-76.6)	13.8 (12.5-15.1)	5.6 (4.7-6.5)	5.7 (4.8-6.6)		
50~59	72.8 (71.1-74.5)	13 (11.7-14.3)	6.1 (5.2-7)	8.1 (7.1-9.1)		
≥60	45 (43.1-46.9)	13.5 (12.2-14.8)	12.6 (11.3-13.9)	28.9 (27.1-30.7)		
Educational level						
illiteracy	49.2 (44.2-54.2)	13.2 (9.8-16.6)	9.3 (6.4-12.2)	28.2 (23.7-32.7)	309.50	<0.001
primary school	57.6 (55–60.2)	14.2 (12.4-16)	9.9 (8.3-11.5)	18.3 (16.3-20.3)		
junior high school	71 (69.5-72.5)	13.5 (12.4-14.6)	5.8 (5–6.6)	9.7 (8.7-10.7)		
senior high school	71.8 (70.3-73.3)	14 (12.9-15.1)	5.7 (5–6.4)	8.4 (7.5-9.3)		
3 year college graduate or above	68.4 (66.9-69.9)	14.5 (13.4-15.6)	7.7 (6.9-8.5)	9.4 (8.5-10.3)		
Population density						
urban	69.8 (68.7-70.9)	13.7 (12.9-14.5)	6.9 (6.3-7.5)	9.6 (8.8-10.4)	7.59	0.055
suburban	70.2 (69.1-71.3)	14.7 (13.8-15.6)	5.9 (5.3-6.5)	9.1 (8.4-9.8)		
Total	70 (69.2-70.8)	14.1 (13.5-14.7)	6.5 (6.1-6.9)	9.4 (8.9-9.9)	-	-

### Demographic variables affecting influenza vaccination uptake

No significant association between gender and vaccination coverage rates existed in any of the three seasons. With respect to the urban-suburban variation in vaccination coverage rates, a significant difference was only noted in 2008/2009 (17.7% vs. 15.6%, p=0.005). We observed significant differences in vaccine coverage by age and level of education during the three seasons. The vaccination coverage rates for the elderly in the three seasons were 36.3%, 46%, and 43.1%, respectively, and the vaccination coverage rates were significantly higher than the respondents aged < 60 years of age (p<0.001). The vaccination coverage rates decreased with increasing levels of education in all three seasons (chi-square test for trend, p<0.001). Older age and level of education achieved were associated with an increased likelihood of reporting vaccination in 2009/2010 and 2010/2011, respectively (Table 3). Vaccination frequency was not significantly associated with gender or population density. The vaccination frequency among the elderly was significantly higher than the respondents < 60 years of age (p<0.001), and decreased with an increased level of education (chi-square test for trend, p<0.001).

**Table 3:** Multiple logistic regression analysis of demographic variables affecting influenza vaccination uptake

	**season 2008/2009**			**season 2009/2010**			**season 2010/2011**		
	**Adjusted**	**95%**	**p value**	**Adjusted**	**95%**	**p value**	**Adjusted**	**95%**	**p value**
	**odds**	**Confidence**		**odds**	**Confidence**		**odds**	**Confidence**	
	**ratios**	**Interval**		**ratios**	**Interval**		**ratios**	**Interval**	
Age (years)									
Age<60	1			1			1		
Age≥60	3.659	3.315-4.037	<0.001	3.865	3.499-4.270	<0.001	5.496	4.984-6.060	<0.001
Educational level									
junior high school or above				1			1		
primary school				1.136	0.996-1.294	0.057	1.252	1.091-1.437	0.001
illiteracy				1.384	1.109-1.727	0.004	1.508	1.201-1.894	<0.001

### Reasons for non-vaccination

Among participants who reported no influenza vaccinations over the previous three seasons, the most commonly reported reason for non-vaccination was ‘I don’t think I am very likely to catch the flu’ (49.3%), and the proportion of other reasons being chosen by respondents was < 20.0% (Table 4). Compared to the elderly, more respondents < 60 years of age reported ‘I don’t think I am very likely to catch the flu’ (50.6% vs. 41.6%; p<0.001), ‘The influenza vaccination is too expensive’ (19.2% vs. 13.8%; p<0.001), and ‘I have no spare time to get vaccination’ (15.8% vs. 13.5%; p=0.001), whereas ‘I am afraid of the side-effects’ was reported more frequently by the elderly than those < 60 years of age (17.4% vs. 15.2%; p=0.022). Compared to the well-educated participants, the illiterate participants were less likely to report ‘I don’t think I am very likely to catch the flu’ and ‘I am afraid of the side-effects’ (p<0.001), whereas ‘The influenza vaccination is too expensive’ and ‘I have never considered it before’ were reported more frequently by the illiterate participants than the well-educated participants (p<0.001; Table 5).

**Table 4:** Reasons for not having vaccination in all three seasons by age

The stated reasons*	**Age (years)**		**χ**^**2**^	**p value**	**Total**
	**Age<60**	**Age≥60**			
	**Weighted % (95% CI)**	**Weighted % (95% CI)**			**Weighted % (95% CI)**
I don’t think I am very likely to catch the flu	50.6 (49.6-51.6)	41.6 (39.3-43.9)	38.33	<0.001	49.3 (48.4-50.2)
I have no spare time to get vaccination	19.2 (18.5-20)	13.8 (12.2-15.5)	22.23	<0.001	18.4 (17.7-19.1)
The influenza vaccination is too expensive	15.8 (15–16.5)	13.5 (11.9-15.2)	11	0.001	15.5 (14.8-16.2)
I am afraid of the side-effects	15.2 (14.4-15.9)	17.4 (15.6-19.1)	5.26	0.022	15.5 (14.8-16.2)
I don’t think the vaccine is effective enough	14.9 (14.2-15.6)	15.3 (13.6-16.9)	0.83	0.362	15 (14.4-15.6)
I don’t think influenza is a serious illness	10.4 (9.8-11)	12.1 (11.5-12.7)	5.51	0.019	10.6 (10–11.2)
I have the specific contraindications	6.6 (6.1-7)	10.7 (9.2-12.2)	48.86	<0.001	7.1 (6.6-7.6)
I have never considered it before	4.5 (4.1-4.9)	8.1 (6.8-9.3)	33.23	<0.001	5 (4.6-5.4)

* Respondents were allowed to state more than one reason.

**Table 5:** Reasons for not having vaccination in all three seasons by level of education

The stated reasons*	**Age (years)**			**χ2**	**p value**
	**illiteracy**	**primary school**	**junior high school or above**		
	**% (95% CI)**	**% (95% CI)**	**% (95% CI)**		
I don’t think I am very likely to catch the flu	28.7 (23.5-33.9)	39.8 (37–42.6)	50.4 (49.4-51.4)	95.07	<0.001
I have no spare time to get vaccination	15.9 (11.7-20.1)	16.4 (14.3-18.5)	18.6 (17.9-19.4)	4.61	0.1
The influenza vaccination is too expensive	25.3 (20.3-30.3)	22.6 (20.2-25)	14.8 (14.1-15.5)	68.14	<0.001
I am afraid of the side-effects	11.1 (7.5-14.7)	11.4 (9.6-13.2)	15.6 (14.9-16.3)	18.77	<0.001
I don’t think the vaccine is effective enough	11.4 (7.7-15.1)	13.1 (11.2-15)	14.5 (13.8-15.2)	3.74	0.154
I don’t think influenza is a serious illness	8.7 (5.5-12)	10.5 (8.8-12.2)	10.1 (9.5-10.7)	0.908	0.635
I have the specific contraindications	10 (6.5-13.5)	9.5 (7.8-11.2)	7 (6.5-7.5)	13.03	0.001
I have never considered it before	12.5 (8.7-16.3)	9.8 (8.1-11.5)	4.5 (4.1-4.9)	91.48	<0.001

* Respondents were allowed to state more than one reason.

## Discussion

The survey consistently showed low rates of influenza vaccination coverage in Beijing in 2008/2009, 2009/2010, and 2010/2011, which were less than the rates from surveys in five European countries in 2006/2007 (25.0% in the UK, 27.4% in Germany, 21.8% in Spain, 24.2% in France, and 24.4% in Italy) [12].

The current study showed that the vaccination coverage was higher during the pandemic. Vaccination has been provided free of charge to the priority populations in each season, and the priority groups for vaccination vary in different seasons. Although the elderly and students are included in each time period, in the pandemic season other populations are also included (e.g., public servants in key positions, teachers, and individuals with chronic diseases) [22]. The phenomenon may partially explain the higher vaccination rate during the pandemic. Furthermore, the coverage in 2009/2010 was calculated by adding the rates for seasonal and pandemic influenza, as the seasonal and pandemic influenza vaccination campaigns were conducted in 2009/2010. The vaccination coverage was nearly the same before and after the 2009 pandemic influenza A (H1N1), and the significantly increased uptake of vaccination during the pandemic was not sustained. Approximately one-half of the respondents reported ‘I don’t think I am very likely to catch the flu’ in the current study. Similarly, a survey in seven urban and two rural areas of China showed that the pandemic had not caused the public to panic [22]. The perception of a low personal risk of threat may partially explain why the 2009 pandemic had no impact on coverage in 2010/2011 [23].

The current study showed that the elderly were more likely to be vaccinated than younger people, and the vaccination coverage rate for the elderly increased after the pandemic. The vaccination coverage rates for the elderly in Beijing increased substantially from 1.7% during 1999–2004 [24] to 43.1% in 2010/2011. However, the current vaccination coverage rates among the elderly in Beijing are significantly lower than recently reported vaccination coverage rates among the elderly living in five European countries (43.1% [95% CI, 44.1-48%] in Beijing 2010/2011 vs. 60.4% [95% CI, 59.4-61.4%] in Europe 2007/2008 [13]); more importantly, these estimates were significantly less than the World Health Organization target level of 75% [12]. The vaccination coverage rate for younger adults in Beijing was even less, increasing from 3.65% during 1999–2004 [24] to < 15% in 2010/2011. Since 2007, seasonal influenza vaccination has been provided free of charge to people ≥ 60 years of age and primary and middle school students in Beijing. The policy of free vaccination may be the main reason for the increasing and higher vaccination coverage rates among the elderly. A study in Hong Kong reported the uptake of vaccination against influenza is sensitive to personal costs, and approximately 45% of the respondents would be highly likely to take advantage of free vaccines [16]. These studies demonstrated that easy access to free vaccination plays a key role in improving the vaccination coverage rates. Another reason for higher vaccination coverage rates among the elderly is that they were less likely to report ‘I don’t think I am very likely to catch the flu’ in the current study, which was the only barrier to influenza vaccination.

A higher level of education is usually thought to be positively associated with vaccination uptake [25]; however, we found that a low level of education had a positive impact on the influenza vaccination uptake. In the current study, illiterate respondents were less likely to indicate that they did not expect to contract influenza compared to those reporting higher levels of education. Second, media broadcasts and internet discussions have fueled social suspicion about the safety and effectiveness of influenza vaccination. People with a low level of education are less likely to be exposed to such information [19], which might have a negative effect on the vaccination coverage rates. In the current study, fear of side effects was less frequently reported by illiterate people. In addition, we found that the elderly reported lower levels of education than younger people. Thus, the free vaccination policies for elderly adults may have also contributed to the higher vaccine coverage among adults with lower education that observed in the current study.

Previous studies have shown that a fear of side effects, doubts about vaccine efficacy, concerns about the danger of influenza, considerations about susceptibility to influenza, and information provided by healthcare professionals can influence the vaccination coverage rate [11,20]. The surveys in five European countries showed that feeling unlikely to contract influenza was the main reason for non-vaccination, and 36% of the respondents felt unlikely to contract influenza in 2006/2007 [12], which was 49.3% reported in the current study. We also found that the proportion of other reasons for non-vaccination chosen by respondents was < 20.0%. The results indicated that the perception of not expecting to contract influenza was the only barrier to influenza vaccination.

These results indicate that different measures should be jointly taken to increase the influenza vaccination coverage rates in Beijing. First, attention should be paid not only to the elderly and students, but also to those groups in which vaccination coverage rates are relatively low (e.g., younger people and well-educated people). Second, we found that the perception of a low risk of threat had a key negative effect on vaccination in this study, so the information about perceptions of personal risk should be delivered to the public via broadcast media when holding a vaccination campaign. Finally, we observed that the influenza vaccination coverage was significantly lower among young people, but free influenza vaccines were not provided to young people. Thus, policy measures should also be undertaken to reduce the financial burden of vaccination for all age groups.

There were some limitations in this study. First, because the questionnaire was self-administered, the respondents recalled their experience from 2008/2009 to 2010/2011, which may have introduced recall bias in data collection. The interviewees might report ‘uptake of vaccination’ because they felt a social pressure by the interviewer. This might partially explain the reported higher uptake among illiterate people. Nevertheless, previous studies have shown that self-reported vaccination data are commonly used in epidemiologic research and are reliable [26]. Second, reasons for non-vaccination may differ from season-to-season, but respondents were not able to provide different reasons for each season on the survey. Nevertheless, the study in five European countries reported nearly the same reasons for non-vaccination from 2002/2003 to 2006/2007 [12,13]. Furthermore, the fact that the response options were shown in a fixed order may impact the results of reasons for non-vaccination. Nevertheless, most of the interviews were undertaken by local health workers who had good relationships with the participants and knew how to motivate the participants. Third, the survey was conducted in January 2011, which was prior to the end of the 2010/2011 season, thus the vaccination coverage rates in 2010/2011 might be underestimated. Nevertheless, the influenza vaccines were provided to the residents from September (or October) to November according to the rules of Beijing Health Bureau (e.g., 20 October to 30 November in 2010/2011) [27], thus very few people received influenza vaccination after December. Fourth, because attitudes regarding vaccination among all respondents were not surveyed in our study, it is impossible to discern whether or not uptake was mainly affected by the perception of low personal risk; it is possible that those who were vaccinated also did not feel at higher risk of infection, but were vaccinated for other reasons.

## Conclusions

This is the first study to assess the influenza vaccination coverage rates in the general population since the policy of free influenza vaccination to the priority populations, including people ≥ 60 years of age and primary and middle school students, was implemented in Beijing. The overall coverage rates were consistently low in recent years, and changed little after the 2009 influenza pandemic. Although the coverage rate for the elderly was significantly higher than in younger people, the coverage rate still did not meet the WHO target level of a 75% vaccination coverage in 2010/2011. The perception of not expecting to contract influenza was the barrier to influenza vaccination. Thus, further measures, such as delivering information about perceptions of personal risk to the public, are needed for vaccination coverage improvement.

## Competing interests

The authors declare that they have no competing interests.

## Authors’ contributions

WS, YP, LH, ZY, and WQ designed the study. WS, LH, and MC performed the data collection. WS analyzed the data and wrote the manuscript. All authors have read and approved the final version of the manuscript.

## Pre-publication history

The pre-publication history for this paper can be accessed here:

http://www.biomedcentral.com/1471-2458/13/636/prepub
